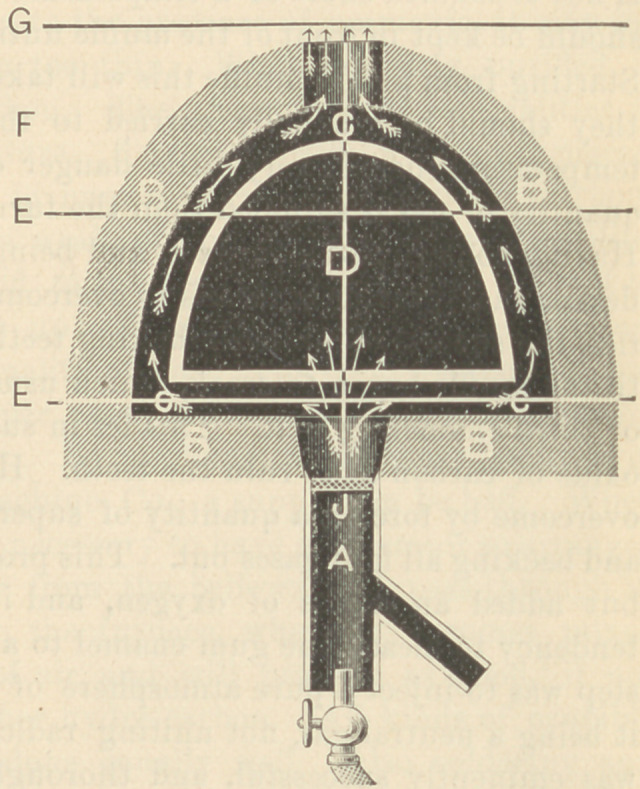# Hydro-Carbon Furnaces for Dental Operations

**Published:** 1886-01

**Authors:** C. H. Land

**Affiliations:** Detroit, Mich.


### HYDRO-CARBON FURNACES FOR DENTAL OPERATIONS.

BY DR. C. H. LAND, DETROIT, MICH.

To be able to fuse the body and enamel of which artificial teeth are composed, in an easy and convenient manner, is a thing the profession has studiously sought after, realizing that, when properly accomplished, the means to elevate prosthetic dentistry from an ordinary mechanical enterprise to one of true art, would be at hand. The mere construction of a furnace after the usual modes has been simple enough, and the question of securing the necessary degree of heat was long ago accomplished. However, the ideal furnace demanded much more. It must possess, not only the capacity of a coal or coke fire, but also accomplish the work in less time, and require but the minimum amount of exertion to operate it. Of the many attempts to produce such, nearly all have failed, owing to technicalities that were not well understood.

After many experiments, and their accompanying failures, it has been demonstrated that to heat an eight-inch muffle, three and one-half by two and one-half inches in diameter, to over 2800° F, represents about a one-man power, equivalent to the exertion of running the ordinary foot-lathe, or the No. 9 bellows, as manufactured by the Buffalo Dental M'f'g. Co., which gives a working pressure of one and one-half pounds to the square inch, and corresponds exactly to the required amount of air pressure and volume necessary to heat an eight-inch muffle to 2800° F. Therefore, to make a furnace larger would require too much power, and one smaller would not do for large pieces of work. In the production of a suitable furnace, the whole working apparatus must be as nearly air-tight as possible, and the supply of gas and air must be easily controlled and well balanced, with the least amount of friction in the passage through the pipes. These, with many minor details, form the basis of a practical gas furnace.

### GASING THE BODY AND ENAMEL.

The most serious trouble with all gas furnaces, has been the extreme liability of injuring the body and enamel by what has been commonly called "gasing." The accompanying illustration will make the philosophy of combustion more clear, and give the reasons why teeth are injured. A, represents the burner; B B B, fire-brick lining; C C C, combustion chamber; D, interior of muffle. The arrows indicate the direction of the blast. The space in the combustion chamber between the lines E E, is where carbon monoxide is formed, a gas containing one equivalent less of oxygen than carbon dioxide, simply an imperfect state of combustion. It is this gas that injures the body and enamel. By reference to the illustration, it will be seen that the little arrows are made to appear passing through the pores of the muffle, and as the direction of the blast from the burner A is directly against the bottom of the muffle, with a pressure of one pound to the square inch, a portion of the carbon monoxide is extremely liable to be forced through its pores, and will be taken up with the body during the first and second biscuiting, here to remain until the enameling process, and as this takes a much higher degree of heat, it causes the gas to be eliminated, as shown in the numerous small bubbles on the surface. The space between the lines E E, and within the combustion chamber C C C, should be known as the first stage of combustion, where a certain portion of carbon monoxide is always present, and the space F, between the lines G and E within the chamber C, should be known as the second stage, which is perfect combustion. In the first stage of combustion one equivalent of oxygen from the atmosphere unites with the hydro-carbon to form carbon monoxide; in the second stage, two, or perhaps three, unite to form carbon dioxide, or carbonic acid. Perfect combustion is always at the extreme point of the blow-pipe, as shown in the illustration.

The attempt, therefore, should be to place the muffle as nearly as possible in the centre of perfect combustion. As carbon monoxide is not consumed short of a temperature of over 2200° F, the teeth should be kept in front of the muffle until it approaches a white heat. Starting from a cold muffle this will take about twelve minutes, and they should be gradually carried to the extreme end. At a high temperature there is very little danger of gasing, unless a greater quantity of gas is supplied than the furnace is capable of burning. Having constructed a furnace, and being familiar with many other details that provide a means to overcome all the apparent difficulties, the success of properly baking teeth seemed to be assured, until the muffle began to crack, which usually started in the second or third enameling heat. This let in such a quantity of the monoxide of carbon as to ruin the teeth. Here was a difficulty that was overcome by forcing a quantity of superheated air into the muffle, and backing all foul gases out. This proved to be a cure for gasing, but added an excess of oxygen, and it was found that this had a tendency to bleach the gum enamel to a lighter shade. The next step was to inject a pure atmosphere of nitrogen into the muffle, it being a neutral gas, not uniting radically with anything. This was eminently successful, and thoroughly demonstrated the fact that porcelain baked in an atmosphere of nitrogen was absolutely perfect, both in color and texture. It therefore gives me pleasure to be able to announce to the profession, that the baking of all kinds of porcelain with any of the hydro-carbons has been brought within the range of every dental practitioner, so that with a little experience and knowledge of the above facts, artificial teeth can be baked, with unerring precision, in a very comfortable, cheap, and easy manner. By a simple attachment, each furnace produces its own nitrogen as fast as needed, and with recent improvements in the construction of muffles and the aid of a small motor, the author has been able to maintain a constant and uniform temperature above 2800° F, by which a slab of sectional gum teeth was completed every seven minutes, at the will of the operator.

### OLEFIANT GAS AND GASOLINE.

Olefiant gas, with which nearly all our cities and towns are supplied, is a compound of hydrogen and carbon. Its symbols are $C^2 H^4$, differing from gasoline only in its specific gravity, the composition of the latter being also $C^2 H^4$. The former will rise to the top of a building, while the latter will fall. The former is more penetrating, therefore more liable to gas the teeth, and hence requires more care in handling. The quality varies in different localities, and sometimes, owing to the presence of ammonia, it may injure the teeth, or it may be too thin. When properly purified it should be a rich hydro-carbon. The uncertainty of its qualities is frequently the cause of failure. To be successful with gas furnaces, it is absolutely necessary to have a pure and rich hydro-carbon. When the gas pressure is weak, or the quality is poor, a gasoline generator may be attached to the pipe and the current allowed to pass through. This takes up a large percentage of the gasoline, and provides a very rich quality of gas. The eighty-seven per cent is the best; seventy-four per cent. is too heavy to use without requiring heat to vaporize it. By applying to the manufacturers of the Combination Gas Machine Co., a supply can be had. When pure gasoline is used, it is necessary to have a generator so arranged that a portion of the air from the bellows will pass through it. This carries the vapor into the furnace, where it becomes mixed with the proper quantity of air, and will produce as good, if not better, results than any other hydro-carbon. All kinds of crucible and muffle work can be done equally well. Also soldering and brazing with the blow-pipe. One gallon of gasoline costs fifteen to twenty cents; this will bake one set of teeth. Therefore, it will be seen that dentists living in localities where there is no gas, will not be deprived of practically the same advantages as their city brethren.

## Figures and Tables

**Figure f1:**